# Psychological well‐being and quality of life in pediatric patients with inflammatory bowel disease in clinical remission

**DOI:** 10.1002/jpr3.70064

**Published:** 2025-07-09

**Authors:** David L. Suskind, Erin Vale, Joy Kawamura, Kendra Francis, Ghassan Wahbeh, Dale Lee, Brooke Musburger, Hengqi B. Zheng, Naomi Schwartz

**Affiliations:** ^1^ Department of Pediatrics, Division of Gastroenterology Seattle Children's Hospital and University of Washington Seattle Washington USA; ^2^ Hawaii Pacific Health Medical Group Honolulu Hawaii USA

**Keywords:** anxiety, Crohn's disease, depression, patient‐reported outcomes measurement information system (PROMIS), pediatric inflammatory bowel disease

## Abstract

**Objectives:**

Pediatric inflammatory bowel disease (IBD) presents significant psychological challenges, including anxiety and depression. This study investigates the prevalence of depression and anxiety symptoms in pediatric IBD patients in remission and examines the correlation between patient and parental assessments of these symptoms.

**Methods:**

Patients with a diagnosis of Crohn's disease (CD), ulcerative colitis, or IBD‐unclassified (IBD‐U) aged 1–21 years in clinical remission as measured by the pediatric CD activity index or pediatric ulcerative colitis activity index were enrolled. Demographic data and patient psychological well‐being were assessed using the IMPACT‐III and the patient‐reported outcomes measurement information system (PROMIS) emotional distress depression short‐form 8a and anxiety short‐form 8a.

**Results:**

Forty‐eight pediatric IBD patients and their parents completed psychological assessments. Prevalence of anxiety and depression were 29.1% and 14.5%, respectively. A moderate inverse correlation between PROMIS depression and anxiety scores, and quality of life was observed. Strong correlations were observed between child‐ and parent‐reported PROMIS scores for both anxiety (*r* = 0.68) and depression (*r* = 0.75).

**Conclusions:**

These findings underscore the persistent burden of anxiety and depression in pediatric IBD patients and the valuable role of parental assessments in identifying psychological distress. Routine screening is essential for improving outcomes in this population.

## Abstract (highlights)

**What is Known:**

- Pediatric inflammatory bowel disease (IBD), including Crohn's disease and ulcerative colitis, is associated with high rates of anxiety and depression.
- Anxiety and depression in pediatric IBD patients negatively impact their quality of life, academic performance, social interactions, and treatment adherence.
- Despite disease remission, pediatric IBD patients remain vulnerable to mental health challenges, underscoring the need for routine screening and multidisciplinary care.

**What is New:**

- Strong correlations were observed between parent and child‐reported patient‐reported outcomes measurement information system scores for both anxiety and depression, supporting the role of parental assessments as reliable proxies for pediatric mental health in IBD.
- Pediatric IBD patients in clinical remission continue to exhibit significant levels of anxiety and depression, highlighting that psychological issues persist even when disease activity is minimal.
- There is a moderate inverse relationship between quality of life (IMPACT‐III scores) and anxiety and depression, suggesting that improvements in psychological well‐being are tied to better health‐related quality of life.

## 1 INTRODUCTION

Inflammatory bowel disease (IBD), comprising of Crohn's disease (CD) and ulcerative colitis (UC), manifests as chronic inflammation of the gastrointestinal tract. IBD is caused by a complex interplay of genetic, environmental, and immunological factors. Most patients are diagnosed in adolescence and young adulthood. The psychological toll of IBD is a significant concern, as the prevalence of anxiety in IBD ranges from 19.1% to 35.1% as compared to 7.1% of the general population.^1^, ^2^ Anxiety and depression appear to have a strong association with IBD disease activity.^2^ In patients with pediatric onset IBD, there is a 50% increased risk of developing depression.^2^

Despite advancements in treatment modalities leading to clinical remission, the burden of mental health challenges persists, often underrecognized and undertreated.^3^ Less is known about the prevalence of psychological challenges among youth with IBD in remission. Understanding the prevalence, risk factors, and impact of depression and anxiety symptoms in pediatric IBD patients, especially during times of clinical remission when these concerns might be more overlooked, is essential for holistic patient care and improved long‐term outcomes.

Children and adolescents with IBD have higher rates of anxiety and depression than their healthy peers, though prevalence rates vary across studies. Various approaches to assessing depression and anxiety in pediatric IBD patients deserve careful consideration. Parents often serve as primary caregivers and observers of their children's well‐being, offering unique insights into the psychological struggles their children may face. Exploring the correlation between parental assessments of depression and anxiety and the patients' own reported levels of these mental health symptoms provides valuable insights. Unfortunately, there is significant discrepancy between patient and parent when it comes to overall IBD symptom reporting in pediatric IBD. Studies show that most pediatric IBD symptom reports offer poor correlation between child and parent symptom scores, with low levels of agreement between children and their parents. On the other hand, parents seem to serve as a good proxy for their children with IBD in the outcome measure of quality of life as measured by the IMPACT III.^4^ However, within this study the degree of concordance between parent and child varied with parents underreporting their child's health‐related quality of life on the IMPACT‐III emotional functioning domain.^4^ To date no study has studied correlations between parent‐ and child‐reports of children's anxiety, depression, and quality of life in pediatric IBD.

This study aims to investigate the burden of depression and anxiety symptoms and their impact on quality of life in pediatric IBD patients in clinical remission as well as parental assessment of patient symptoms. By synthesizing evidence from different perspectives, we seek to gain a better understanding of the psychological landscape in pediatric IBD. To accomplish this, we examined the correlation between parent and child patient‐reported outcomes measurement information system (PROMIS) anxiety and depression in conjunction with IMPACT III scores for IBD‐related quality of life.^5^, ^6^, ^7^

## 2 METHODS

Patients were recruited from Seattle Children's Outpatient IBD Center. Families were approached consecutively during routine clinic visits at the Seattle Children's IBD Center between January 2020 and December 2023. Eligible participants were identified through the electronic medical record and approached in person. Patients with a diagnosis of CD, UC or IBD‐unclassified (IBD‐U) aged 1–21 years in clinical remission as measured by a pediatric Crohn's disease activity index (PCDAI) score of less than 10 or pediatric ulcerative colitis activity index (PUCAI) score of less than 10 for at least 6 months without evidence of acute inflammation and/or elevated acute phase reactant as measured by C‐reactive protein (CRP) < 0.8 mg/dL, or erythrocyte sedimentation rate (ESR) < 20 mm/h within 8 weeks were recruited for this study.^8^, ^9^ Exclusion criteria included active IBD, hospitalization or surgery planned within 3 months, recent medication changes within 8 weeks before enrollment, and other serious medical conditions, such as neurological, liver, kidney, or systemic disease.

### 2.1 Ethics statement

Seattle Children's Hospital Institutional Review Board (CR00007913) approved the study.

### 2.2 Patient characteristics

Demographic data and disease characteristics collected included age at diagnosis and at study enrollment, gender, race, ethnicity, disease type, disease location, and medications. Patient psychological well‐being was assessed using the IMPACT‐III Questionnaire and the PROMIS emotional distress depression short‐form 8a (PROMIS depression) and anxiety short‐form 8a (PROMIS anxiety).^5^, ^6^ The IMPACT‐III questionnaire is a self‐report measure of health‐related quality of life among pediatric IBD patients that contains 35 items across six domains (bowel symptoms, systemic symptoms, social functioning, body image, treatment/interventions, and emotional functioning). The score ranges from 35 to 175, with higher scores indicating a better quality of life. The PROMIS depression and anxiety questionnaires include eight questions related to psychological symptoms in the past 7 days. The PROMIS questionnaires use a T‐score metric in which 50 is the mean of a relevant reference population and 10 is the standard deviation (SD) of that population. A higher PROMIS T‐score represents more severe symptoms. Parents completed the proxy versions of the PROMIS questionnaires.

Descriptive statistics were prepared for all demographic, clinical, and laboratory measures including frequencies and percentages for categorical variables and means, standard deviations, and ranges for continuous variables. Pearson correlation coefficient was calculated to measure the strength and direction of the linear relationship between parent‐proxy and child‐report PROMIS scores, as well as between child‐report PROMIS scores and IMPACT score. All statistical analyses were conducted using R 4.3.2.

## 3 RESULTS

A total of 183 families were approached, and 48 patient‐parent pairs consented and completed the baseline surveys, giving a response rate of 26.2%. There were 48 patient‐parent pairs with PROMIS scores. The average age at diagnosis of included patients was 10.7 years, and a majority were male, had CD, and were non‐Hispanic and White (Table 1). For CD, the most common location of disease was L3 (47.5%) followed by L1 (26.3%).^10^ For UC, the most common location was E4 (86.6%).^10^ Most patients (78.0%) were receiving biologic therapy. Forty‐one pairs of patients and parents had PROMIS scores recorded at 1 year.

**Table 1:** Baseline patient demographic and clinic characteristics.

	All patients
	(*N* = 48)
Sex, *n* (%)	
Female	19 (39.6%)
Male	29 (60.4%)
Disease classification, *n* (%)	
CD	38 (79.1%)
UC	8 (16.7%)
Indeterminant colitis	2 (4.2%)
Race, *n* (%)	
White	36 (75%)
Black/African American	1 (2.1%)
Asian	3 (6.3%)
Mixed	6 (12.5%)
Other	1 (2.1%)
Unknown	1 (2.1%)
Ethnicity, *n* (%)	
Non‐Hispanic	40 (83.3%)
Hispanic	6 (12.5%)
Unknown	2 (4.2%)
Age at diagnosis (years), mean (SD)	10.7 (2.93)
Age at enrollment (years), mean (SD)	14.3 (2.5)
Age range (years)	7–19
Date of enrollment	July 16, 2020–December 11, 2023
Disease location, *n* (%)	
CD	
L1: Distal 1/3 ileal +‐limited cecal disease	12 (28.6%)
L2: Colonic	9 (21.4%)
L3: Ileocolonic	20 (47.6%)
L4a: Upper disease proximal to ligament of treiz	1 (2.4%)
UC	
E1: Ulcerative proctitis	1 (12.5%)
E2: Left‐sided UC	1 (12.5%)
E4: Pancolitis	6 (75%)
Treatment strategy, *n* (%)^a^	
Biologics	38 (66.7%)
Immunomodulators	10 (17.5%)
Specific carbohydrate diet	7 (12.3%)
Surgical intervention/resections	0 (0%)
Other	1 (1.8%)
None	1 (1.8%)
PCDAI score at enrollment, mean (SD)	0.75 (1.69)
PCDAI score at 1 year, mean (SD)	2.5 (4.27)
PUCAI score at enrollment, mean (SD)	1.88 (2.48)
PUCAI score at 1 year, mean (SD)	3.13 (1.78)

Abbreviations: PCDAI, pediatric Crohn's disease activity index; PUCAI, pediatric ulcerative colitis activity index; SD, standard deviation; UC, ulcerative colitis.

^a^ Treatments are not mutually exclusive.

**Figure 1:**
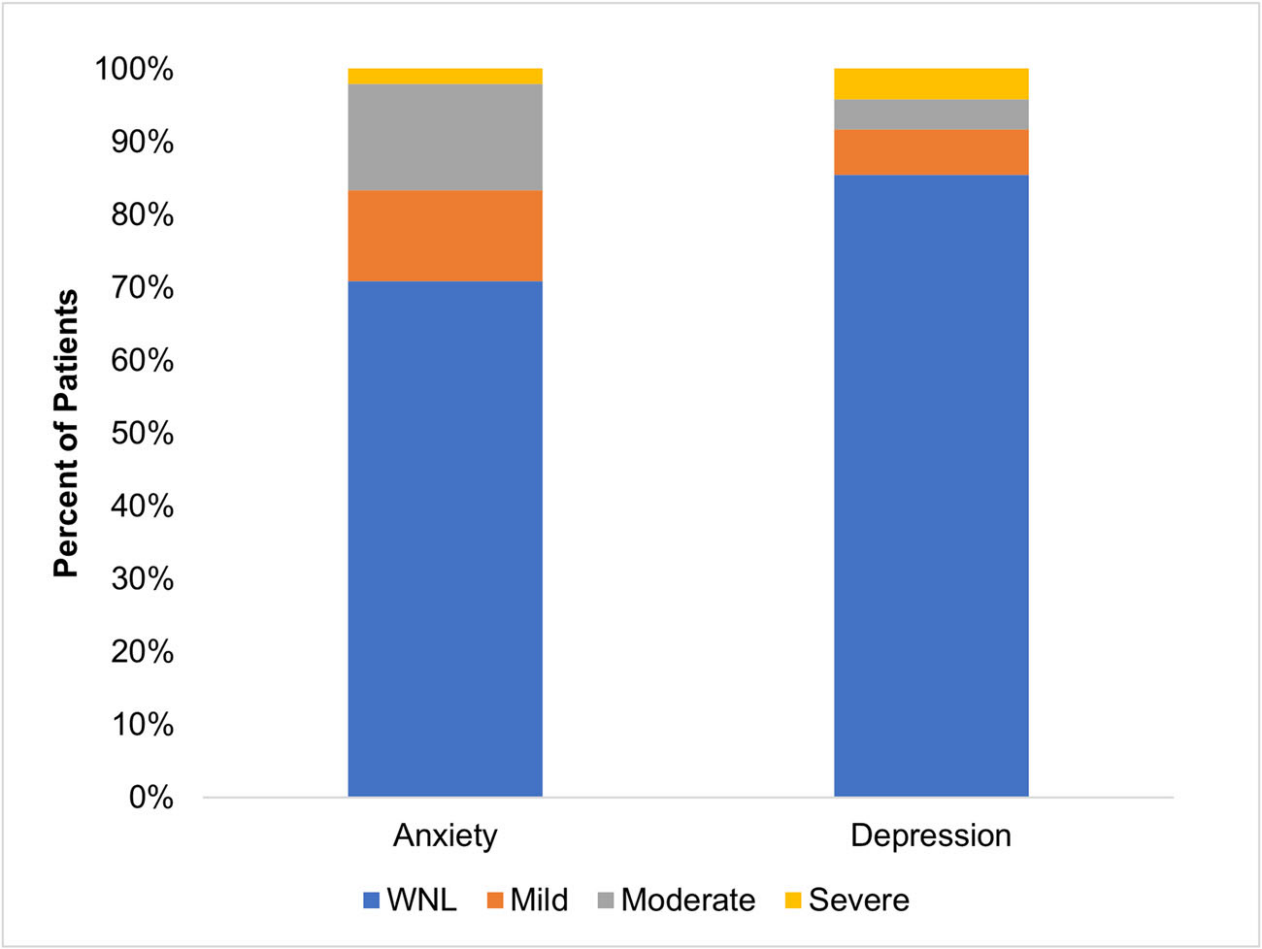
Percent of patients in each category of anxiety or depression per PROMIS cut‐offs. PROMIS, patient‐reported outcomes measurement information system.

**Figure 2:**
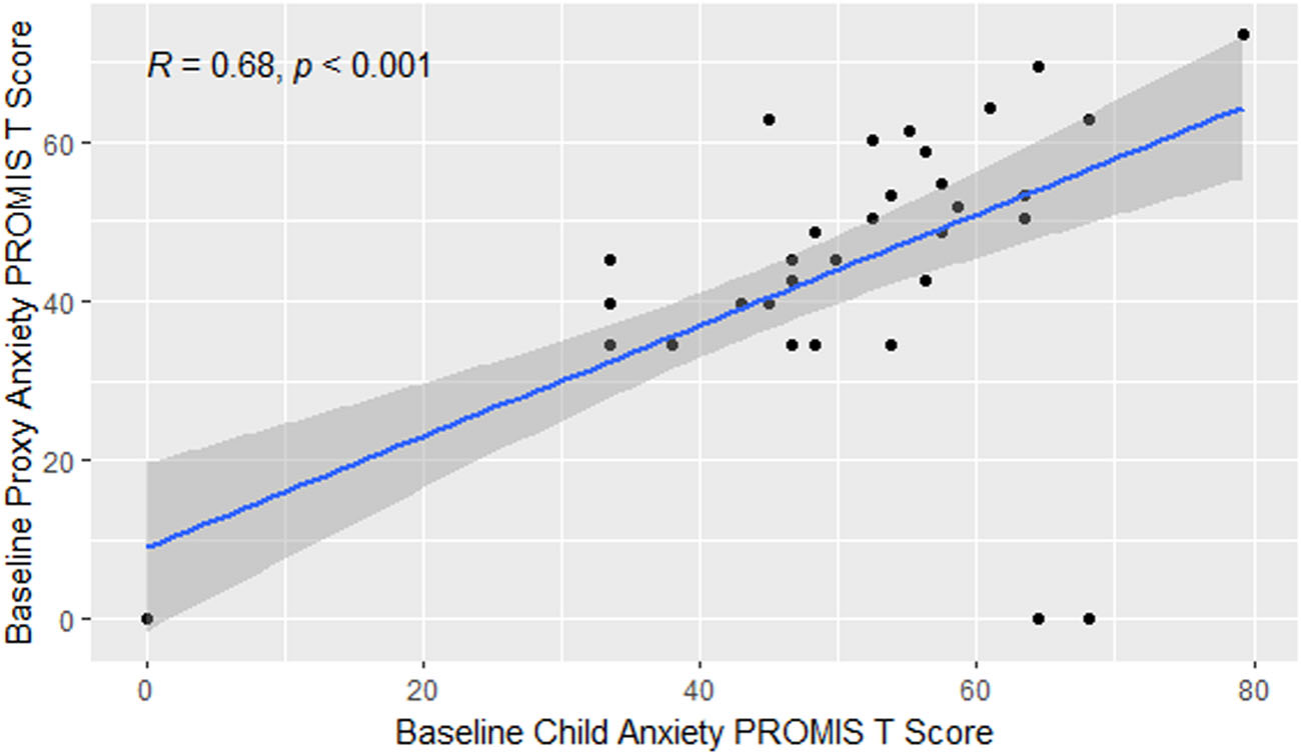
Pearson correlation between child and proxy anxiety PROMIS T scores. PROMIS, patient‐reported outcomes measurement information system.

**Figure 3:**
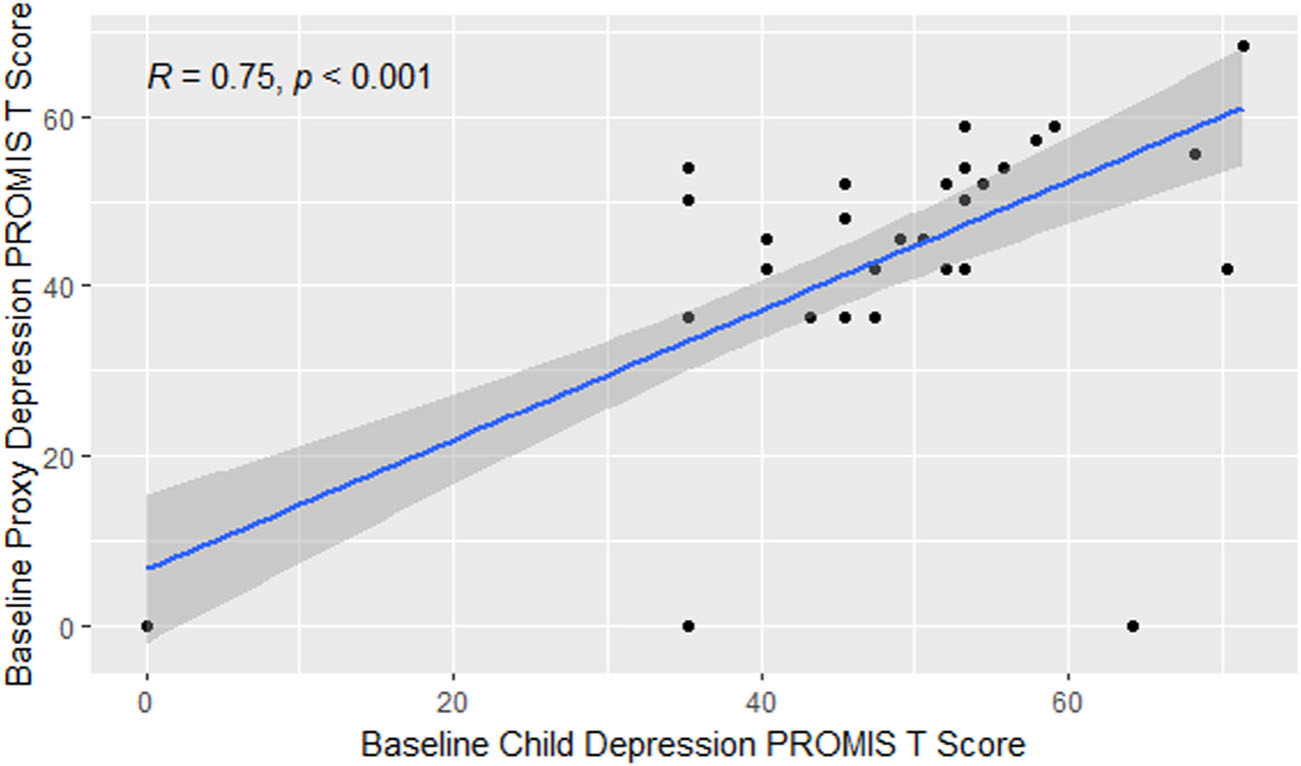
Pearson correlation between child and proxy depression PROMIS T scores. PROMIS, patient‐reported outcomes measurement information system.

Prevalence of anxiety symptoms was 29.1% and depression symptoms were 14.5%, respectively (Figure 1). There was a strong correlation between child and parent PROMIS scores: *r*(46) = 0.68 (95% confidence interval [CI]: 0.49, 0.81) and *r*(46) = 0.75 (95% CI: 0.60, 0.85), for anxiety and depression, respectively (Figures 2 and 3).

Forty‐six patients had both IMPACT and PROMIS scores. Average IMPACT score at enrollment was 77.2 (95% CI: 73.4, 80.9). There was a moderate inverse correlation between IMPACT score and both PROMIS anxiety and depression scores: *r*(44) = −0.62 (95% CI: −0.77, −0.40) and *r*(44) = −0.53 (95% CI: −0.71, −0.29), respectively.

## 4 DISCUSSION

Anxiety and depression often accompany pediatric IBD, significantly affecting both physical health and emotional well‐being.^11^ These issues can easily be overlooked when patients are in remission, despite their impact on quality of life. Accordingly, the International Organization of Inflammatory Bowel disease (IOIBD) recently emphasized the importance of prioritizing quality of life and the absence of disability in treating IBD as long‐term goal of care in the STRIDE2 recommendations for long term target‐to‐treat strategies.^12^

Our research affirms the persistent prevalence of anxiety and depression in pediatric IBD patients, even during periods of clinical remission. These symptoms correspond to patient quality of life, highlighting the need for proactive measures in evaluating and managing mental health in pediatric IBD. Additionally, caregivers exhibit adeptness in identifying anxiety and depression in their children. This underscores the valuable role of parents as proxies for patients when evaluating for anxiety and depression in IBD. When deciding whether to utilize patient or parent proxies, it is crucial to consider the perspectives of both the child and the potential impacts of proxy usage. While pediatric self‐reporting should be the standard for measuring patient‐reported outcomes, there are instances when the child may be unwilling or unable to provide accurate self‐assessment. Although information obtained from proxy‐reporting may not be identical to patient assessment, its correlation to patient assessment is strong enough to add insight into the patient's mental status. In addition, parental/guardian perspectives can offer valuable insights independently related to healthcare utilization, risk factors, and the quality of care provided. While it is important to respect the privacy as well as the autonomy of adolescent patients, it is important at the same time to engage parents to ensure the quality of screening and to capture any psychological challenges.

Pediatric patients with IBD experience higher rates of anxiety and depression compared to their healthy peers, with varying prevalence noted in the literature.^13^, ^14^, ^15^, ^16^ It has been suggested that youth with more established disease and less active disease may be at lower risk for anxiety and depression.^17^ Our sample of youth with IBD in clinical remission displayed significant levels of both anxiety and depression symptoms. This emphasizes the importance that all pediatric patients with IBD are vulnerable to the impact of stress on psychosocial functioning, and thus should be regularly screened for depression and anxiety regardless of disease status. Anxiety and depression not only exacerbate disease symptoms but also impede treatment adherence, academic performance, and social interactions, creating a detrimental cycle.^18^, ^19^, ^20^ In quiescent disease, patients under conditions of low stress and who scored low on avoidance coping (i.e., did not engage in social diversion or distraction) were least likely to relapse, emphasizing the importance of a biopsychosocial model for patient care.^21^ Early detection and intervention through regular screening are pivotal in managing anxiety and depression in pediatric IBD. Collaborative care involving gastroenterologists, pediatricians, and mental health professionals can help to ensure comprehensive support for IBD patients and their families.

This study has several limitations including a relatively small sample size and single center recruitment, which may affect the generalizability of the findings to broader populations with different demographic. Although efforts were made to engage a diverse patient population, Seattle's patient demographics are predominately white. In addition, participation required both patient and parental consent, which may introduce self‐selection bias. The surveys were also only provided in English which further limits the generalizability of the findings. Moreover, we lack the data on the prevalence of anxiety and depression before IBD diagnosis, preventing us from understanding the temporal relationship between mental health symptoms and IBD diagnosis. The reliance on limited data of self‐reported measures such as the IMPACT‐III questionnaire and the PROMIS depression and anxiety questionnaires may introduce bias, as responses can be influenced by individual interpretation and the current psychological state of the patient. Yet, these are validated instruments which can be valuable tools for screening.

Despite these limitations, this study underscores a crucial area for intervention in the care of patients with quiescent IBD and emphasizes the need for vigilance among care providers in addressing psychological issues in these patients. The findings have significant implications for clinical practice, including the integration of routine screening for depression and anxiety, the implementation of multidisciplinary interventions, and the development of personalized treatment approaches tailored to the unique needs of pediatric IBD patients. This study emphasizes the necessity of comprehensive care models that address the holistic needs of these vulnerable individuals, fostering resilience and enhancing overall well‐being.

## 5 CONCLUSION

In conclusion, this study highlights the persistent prevalence of anxiety and depression among pediatric IBD patients, even during periods of clinical remission, and underscores the significant impact these mental health challenges have on quality of life. The strong correlation between parent and child assessments emphasizes the value of incorporating both perspectives in the clinical evaluation of psychological well‐being. Regular screening for depression and anxiety, regardless of disease status, is crucial for improving patient outcomes and ensuring comprehensive care. Ultimately, a biopsychosocial approach to pediatric IBD care, involving early detection, intervention, and collaboration among healthcare providers, is essential for addressing the mental health needs of these patients. Further study should build upon these initial findings and incorporated qualitative study to further understand the nuances of anxiety and depression in this patient population.

## CONFLICT OF INTEREST STATEMENT

David L. Suskind who consults for Nestlé HS and Pharming LLC. The remaining authors declare no conflicts of interest.
